# SOCIAL ISOLATION AND COGNITIVE FUNCTIONING AMONG OLDER JAPANESE ADULTS

**DOI:** 10.1093/geroni/igz038.3168

**Published:** 2019-11-08

**Authors:** Shohei Okamoto, Erika Kobayashi, Jersey Liang

**Affiliations:** 1 Keio University, Minato-ku, Tokyo, Japan; 2 Tokyo Metropolitan Institute of Gerontology, Tokyo, Japan; 3 University of Michigan, Ann Arbor, Michigan, United States

## Abstract

This research aimed to assess the relationship between social isolation and cognitive functioning among older Japanese adults, thereby expanding the relevant literature in two main ways. First, we estimated a social isolation score to incorporate objective measurements of social isolation into a subjective measurement. Second, a panel data analysis was utilised to consider the change in the social isolation score and time-invariant unobserved heterogeneity. Data were derived from the National Survey of the Japanese Elderly, a survey of a sample of older Japanese adults aged 60 to 99 in waves 3 through 7, which contain unified information of social isolation. The sample included 4,889 observations (1,836 individuals) for men and 6,621 observations (2,433 individuals) for women. The predicted isolation score was obtained by a random-effects ordered logistic regression (i.e., regressing a subjective feeling of isolation on variables regarding social interaction, social support, and social engagement). The association of cognitive functioning with the isolation score was estimated by a fixed-effects ordinary least squares regression, controlling for age, socioeconomic variables, health conditions, and time fixed-effects. We found that increased isolation was associated with a deterioration in cognitive functioning, both for men (coefficient: 0.66, robust standard error [SE]: 0.30) and women (coefficient: 0.90, SE: 0.26). Findings of this research highlight the importance of actions aimed at inhibiting social isolation for the prevention of cognitive decline. This approach is potentially beneficial for developing measurements of both subjective and objective social isolation and estimating the longitudinal relationship between social isolation and cognitive functioning.

